# Making sense of conducting a critical interpretive synthesis: A scoping review

**DOI:** 10.1017/rsm.2025.10041

**Published:** 2025-10-08

**Authors:** Saritte Perlman, Eliana Ben-Sheleg, Moriah E. Ellen

**Affiliations:** 1 Department of Health Policy and Management, Ben Gurion University of the Negev, Beersheva, Israel; 2 Institute for Health Policy, Management and Evaluation, University of Toronto, Toronto, ON, Canada

**Keywords:** critical interpretive synthesis, methodology, process mapping, scoping review

## Abstract

Critical interpretive synthesis was introduced in 2006 to address various shortcomings of systematic reviews such as their limitations in synthesizing heterogeneous data, integrating diverse study types, and generating theoretical insights. This review sought to outline the methodological process of conducting critical interpretive syntheses by identifying the methods currently in use, mapping the processes that have been used to date, and highlighting directions for further research. To achieve this, a scoping review of critical interpretive syntheses published between 2006 and 2023 was conducted. Initial searches identified 1628 publications and after removal of duplicates and exclusions, 212 reviews were included in the study. Most reviews focused on health-related subjects. Authors chose to utilize the method due to its iterative, inductive, and recursive nature. Both question-based and topic-based reviews were conducted. Literature searches relied on electronic databases and reference chaining. Mapping to the original six-phase model showed most variability in use of sampling and quality assessment phases, which were each done in 50.7% of reviews. Data extraction utilized a data extraction table. Synthesis involved constant comparison, critique, and consolidation of themes into constructs, and a synthesizing argument. Refining critical interpretive synthesis methodology and its best practices are important for optimizing the utility and impact and ensuring findings are relevant and actionable for informing policy, practice, and future research.

## Highlights

### What is already known?

- Critical interpretive synthesis (CIS) was designed to address limitations of systematic reviews, particularly for synthesizing heterogeneous data.
- CIS emphasizes flexibility and iterative processes, allowing refinement of research questions and synthesis of diverse qualitative, quantitative, and theoretical evidence.

### What is new?

- The study identifies trends, gaps, and best practices across 212 CIS from diverse disciplines conducted between 2006 and 2023.
- Recommendations are provided for standardizing CIS methodologies, including practical steps for sampling, quality appraisal, and data synthesis.

### Potential impact for RSM readers

- This work provides a descriptive overview of current practices in CIS which may inform future efforts to enhance methodological rigor, transparency, and replicability.
- This review underscores how CIS can be adapted to various contexts from health services research to social sciences, fostering interdisciplinary innovation.
- This review provides guidelines for conducting CIS, offering a foundation for future methodological development and refinement, and enhancing the utility of CIS for policy, practice, and theoretical advancement.

## 1 Introduction

A critical interpretive synthesis (CIS) is a type of research synthesis that expands upon standard systematic review methods by utilizing interpretive methods to synthesize different types of evidence.^1^ CIS was originally introduced by Mary Dixon-Woods in 2006 to address some of the various shortcomings of systematic reviews. A critical interpretive synthesis is an applicable research method in situations that require theorization of evidence or where the literature demands a critique of underlying assumptions.^2^ While a traditional systematic review focuses on a pre-established, fixed research questions and selection methods, a CIS evolves from a compass question which can evolve in a transparent manner allowing the literature search and article selection process to evolve as well. Similarly, a CIS applies relevance ratings to the data rather than qualitative ratings. Data are also typically extracted from a purposive sample using an analytic and explanatory framework as opposed to a pre-established extraction and synthesis approach being applied to all studies in traditional systematic reviews.^3^

Since Dixon-Woods’ seminal paper, no codified methodology has been proposed to guide authors undertaking critical interpretive syntheses. While the search and inclusion criteria for conducting a CIS are relatively clear as they are similar to the methods used in a systematic review, methods diverge at the study selection, and data extraction and synthesis stages. A more recent review examined reporting practices, utilization and applicability of critical interpretive syntheses, and identified wide variation in CIS methods and reporting standards.^4^ By evaluating and assessing CIS reporting practices, their work helps advance the use of CIS as a method for synthesizing research findings while ensuring transparency, replicability, trustworthiness, and rigor. Although the authors proposed a hierarchy of key features for standardization of reporting practices, they did not present methodological guidelines for undertaking a CIS. A methodological gap remains as well as criteria to determine when CIS is most appropriate. Given the absence of a methodological guidance for conducting CIS, we sought to outline the CIS methodological process by identifying the CIS methods currently in use, mapping the processes that have been used to date, and highlighting directions for further research.

## 2 Methods

We conducted a scoping review of CIS studies. The study was guided by the framework proposed by Arksey and O’Malley^5^ and reporting follows the Preferred Reporting Items for Systematic Reviews and Meta-Analyses extension for Scoping Reviews (PRISMA-ScR) Checklist.^6^ The protocol was registered on the Open Science Framework (OSF) platform and is publicly available at: https://osf.io/5trmh (Registration number: 5trmh).

To guide the design and analysis of this scoping review, we adopted the six-phase framework for CIS developed by Dixon-Woods et al.^1^ This framework includes: (1) formulating the review question, (2) literature search, (3) sampling, (4) determination of quality, (5) data extraction, and (6) interpretive synthesis. It was selected because it developed and introduced the method and provides the most comprehensive and widely accepted structure for conducting CIS, aligning with the iterative, inductive, and interpretive nature of this methodology. Structuring our research and mapping our findings to these phases allowed us to systematically assess how CIS has been applied across studies and to identify methodological trends and inconsistencies.

### 2.1 Literature search

We conducted a systematic literature search of CIS literature published from its inception in 2006 through December 19, 2023. We used the term “critical interpretive synthesis” and conducted a title and abstract search in the JSTOR, PubMed, EMBASE, CINAHL, Web of Science, PsycINFO, Cochrane Library, and WorldCat electronic databases. All eligible articles were uploaded into Covidence (Covidence systematic review software, Veritas Health Innovation, Melbourne, Australia), and duplicates were identified and removed. For inclusion, articles had to be published after 2006 when the term “critical interpretive synthesis” was coined by Dixon Woods et al.^1^ and employ a critical interpretive synthesis. Only articles published in English were included in the review as translation and verification of non-English CIS papers was not feasible within the scope of this project. Articles were included irrespective of where the research was conducted. Articles were excluded if the full text could not be obtained or if the study did not provide sufficient methodological detail describing how the review was conducted across key stages of the CIS process. Studies that described only some stages were included if they provided enough information about the methods used to allow for comparison and analysis within the review.

### 2.2 Study selection

The initial screening of titles and abstracts was conducted independently by two researchers. The full text of potentially relevant documents identified by either reviewer was obtained for closer examination and review by both researchers. Disagreements were resolved through discussion or by consulting a third author.

### 2.3 Data extraction

A data extraction form was created to extract information related to the (a) manuscript details (e.g., study authors, title, year of publication); (b) research setting (e.g., country and research context); (c) characteristics and methodology according to Dixon-Woods et al.^1^; (d) factors associated with the perceived quality of strategies (e.g., limitations, strengths, and appropriateness considerations); (e) reporting elements (e.g., PRISMA flow chart and prior publication of a protocol) (Supplementary Material 1). Prior to full data extraction, the data extraction form was piloted independently by each researcher with five of the references to determine the clarity of the form to ensure consistency and accuracy of data extraction. Data extraction was conducted by one researcher and confirmed by a second researcher. Disagreements were resolved by discussion or consulting a third study author. Frequency analysis was used to report study characteristics. Methodological strategies used in the reviews were mapped according to the six-phase model in Dixon-Woods et al.’s seminal paper.

## 3 Results

**Figure 1:**
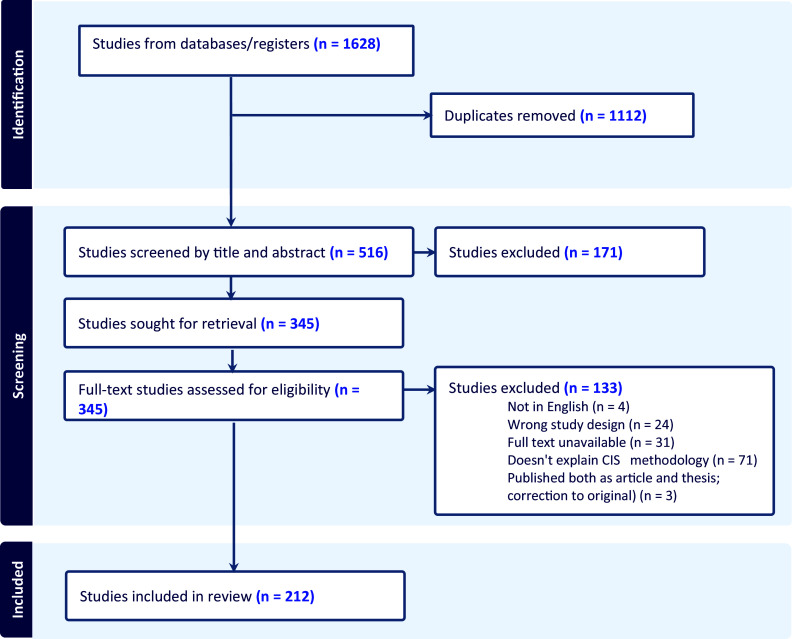
PRISMA.

**Figure 2:**
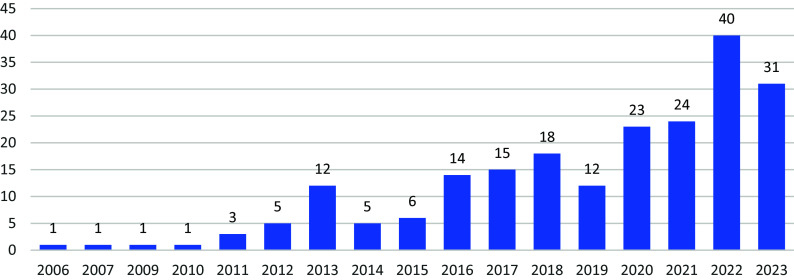
Publication of CIS by year.

We identified 1628 publications. After removal of 1112 duplicates, 171 were excluded at the title and abstract stage and 133 after full-text screening (Figure 1). We included 212 CIS reviews published between 2006 and 2023 that met our inclusion criteria (Supplementary Material 2). A growing trend in the use of this methodology could be noticed with only four studies conducted from 2006 to 2010, compared to a minimum of 23 CIS published per year since 2020 (Figure 2).

The most CIS were produced in the United Kingdom (*n* = 66), followed by Canada (*n* = 50), Australia (*n* = 32), and the United States (*n* = 29). Included CIS reviews were published in 131 different journals, 17 PhD theses that employed CIS were also included; 85.4% (*n* = 181) of CIS did not publish their study protocol prior to publishing their research. Protocols were considered published if the article explicitly stated this and provided a link or registration details (e.g., PROSPERO, OSF). The majority of CIS reviews were carried out in the context of clinical health services, healthcare delivery, policy and research, and health equity and public health. Other research contexts included education, music therapy, social work and policy. The number of studies included in the CIS ranged from 7 to 267, 179 CIS included <100 studies, 17 CIS included 101–200 studies, 8 CIS included >200 studies, 5 studies did not cite how many articles were included. The most common number of included studies were 10 and 40 (*n* = 8 CIS), 13, 20, 27 (*n* = 7 CIS each); 65.6% of CIS utilized a PRISMA flow diagram to illustrate their search and inclusion results.

### 3.1 CIS reasoning

The most frequently cited reasoning for utilizing this methodology was that CIS allows for the inclusion and integration of data from methodologically diverse studies, such as qualitative, quantitative, mixed methods studies, and theoretical papers. Furthermore, through the induction and interpretation of the data and the creation of synthetic arguments, CIS seeks to produce new concepts, enable the emergence of a new understanding of the literature, and offer recommendations for practice. Articles stated that CIS is useful for reviewing a novel field and creating theoretical frameworks. CIS was characterized as a dynamic, flexible, and iterative review process. An additionally frequently cited reason for choosing CIS was that it is suited to deal with a heterogeneous body of literature that cannot be effectively reviewed and analyzed using more conventional approaches, such as systematic reviews and meta-analyses. CIS allows for flexibility in the synthesis of an amalgamous, conjectural, and discursive body of literature, development of a theoretical account, and practical application with potential for insights to otherwise complex questions.

Limitations of conducting a CIS included the quality of reporting between included articles, the focus of existing literature on more diverse objectives which limits ability to address the CIS research question directly, searches did not aim to capture all relevant literature therefore data from omitted sources could have added new dimensions to generated theories, the interpretive and creative nature of the review which leads to subjectivity, reproducibility, and transparency difficulties.

### 3.2 Methodological elements to conducting a CIS

#### 3.2.1 Dixon-Woods phase 1: Review question

Conducting a review begins with formulation of a review question, in the case of CIS the research question is not intended to be specified and precise at the outset of the review with the formation of a cohesive, grounded question; 60.4% (*n* = 128) of CIS were question based, 38.7% (*n* = 82) were topic based, and 0.9% (*n* = 2) used both questions and topics. Among topic-based CIS (*n* = 82), 51.2% (*n* = 42) had one broad topic or did not specify additional subtopics, 28.0% (*n* = 23) had 2 topics, 7.3% (*n* = 6) had 3 topics, and 4.9% (*n* = 4) had >3. Among the question-based CIS, 44.5% (*n* = 57) had 1 question, 13.3% (*n* = 17) had 2 questions, 13.3% (*n* = 17) had 3 questions, 4.7% (*n* = 6) had 4 questions, 1.6% (*n* = 2) had 5 questions, and 3.9% (*n* = 5) had one main question and a series of subquestions. While Dixon-Woods et al. referred to these guiding questions/topics as “a compass” question, only 9.4% (*n* = 20) CIS in this study utilized the term, of which 7.1% (*n* = 15) had a compass question and 2.3% (*n* = 5) had a topic as their compass.

#### 3.2.2 Dixon-Woods phase 2: Literature search

Almost all of the CIS reviews employed database searches to find relevant papers, searching one to eight databases utilizing search techniques comprising keywords connected together using Booleans to locate these keywords in titles, abstracts, and where accessible, subject headings. Authors may have sought advice from a university librarian or information specialist knowledgeable about electronic database search strategies. The next most popular strategy for searching the literature was hand searching reference lists. Citation snowballing was occasionally used to identify additional relevant studies. Other methods included contacting experts, well-known researchers in the subject and stakeholders, getting advice from professionals, getting recommendations from colleagues in the field, and using researchers’ own expertise to find pertinent material from related fields that was not immediately or obviously related to the question. Hand search of key journals, such as the top 10 journals in each field, and book chapters was also used. In addition, searching websites of organizations with known repositories of gray literature, such as international and national nongovernmental organization websites, was used in several CIS reviews. Gray literature was included as sources for analysis and synthesis, and sources to provide contextual information regarding the data.

Regarding flexibility in relation to a search, CIS allows search strategies to emerge as part of the review process and as such some CIS reviews conducted preliminary searches, prior to determining whether a structured search served the needs of the research questions. Once relevant records were identified, and duplicates were removed, inclusion and exclusion criteria were used for screening and full-text reviews for selection processes. Inclusion and exclusion criteria were applied during the initial screening and full-text review stages to determine eligibility based on predefined methodological and topical relevance. Eligibility assessments for content were made based on how identified records addressed the existing theory and conceptual bases, researchers’ desire to explore particular elements, relevance to the research questions, contribution to understanding or action, introduced a novel or unique perspective, or contributed to theory generation.

Many CIS studies applied inclusion and exclusion criteria during the literature search phase, mirroring conventional systematic review practices. This approach was not always clearly distinguished from conceptual sampling. In several cases, authors conflated systematic screening with purposive sampling, reflecting the methodological ambiguity present in the field. In contrast to systematic screening, conceptual sampling in CIS is intended to be a process to focus on theoretical and conceptual relevance rather than a data summary. This was inconsistently reported across studies. Some authors used the term “sampling” to describe what appeared to be standard inclusion/exclusion procedures, while others did not distinguish between the two at all. We have therefore interpreted and reported these phases with attention to how they were described in the original studies, while acknowledging the overlap.

#### 3.2.3 Dixon-Woods phase 3: Sampling

A formal sampling process was included in 50.5% (*n* = 107) of CIS reviews. Some CIS reviewed only key articles or samples of four to five of the most relevant articles, while others reviewed all of the included studies. Among the reviews that did sample the studies, purposive, theoretical, and representative sampling was used to select studies based on relevance and theoretical contribution, which evolved with theory generation until theoretical saturation was reached. The goal of using this method was to maximize the relevance and theoretical contribution of the included papers. Some CIS reviews used multiple sampling methods to select papers, augment the literature search, fill in knowledge and conceptual gaps, enhance and elaborate emerging analyses, and explore the consistency and generalizability of hypotheses. Some CIS reviews used the term “sampling,” but subsequently described screening and article selection processes. Sampling and selection were driven by conceptual relevance, whereas authors sought to maximize the range of articles identified as important rather than to accumulate multiple mentions of more diverse yet similar concepts.

#### 3.2.4 Dixon-Woods phase 4: Determination of quality

CIS reviews were evaluated to see if they used a quality assessment or not, and if so, whether it was done before or after data extraction, and whether studies were excluded from the analysis and synthesis based on their quality; 50.5% (*n* = 107) studies reported performing a quality assessment, however, they varied greatly in the quality assessment tools they used in their entirety, modified, or in combination with other tools. The original CIS advised only excluding “fatally flawed” articles and focusing on the relevance rating rather than the rigor. Relevance was embedded in the inclusion and exclusion decisions reported by authors as an element of selecting articles based on conceptual contribution, theoretical richness, or alignment with the evolving synthesis. For CIS reviews that did conduct formal quality appraisal, the Dixon-Woods et al. quality appraisal checklist^1^ was used in 18 studies, various Critical Appraisal Skills Programme (CASP) quality assessment tools^7^ in nine studies, checklists from the Joanna Briggs Institute (JBI)^8^ in six studies, the Jadad scale for quantitative articles^9^ in four studies, the Hawker Quality Assessment tool^10^ in four studies, and the mixed method appraisal tool (MMAT)^11^ in three studies. Other tools cited were, modified Cochrane Netherlands guidelines,^12^ Crowe Critical Appraisal Tool (CCAT),^13^ Authority, Accuracy, Coverage, Objectivity, Date, and Significance (AACODS) checklist,^14^ NICE quality appraisal checklists^15^ ^,^ ^16^ and reporting guidelines such as the COREQ checklist,^17^ Strengthening the Reporting of Observational Studies in Epidemiology (STROBE) checklist,^18^ and GRADE-CERQual checklist,^19^ each used in only one study. The remaining 56 studies did not specify the tools they used for quality appraisal. Quality assessment was intentionally not performed in 33 studies, while in 65 studies it was unclear. In 12 studies, the quality assessment was reported to be performed prior to data extraction, while in 4 studies, it was performed after data extraction. Nineteen studies reported that studies were excluded from the analysis and synthesis based on the quality assessment due to being fatally flawed, of low quality, or failing to pass a certain score. Twenty studies reported that they included all papers, regardless of quality, because studies with low methodological standards can also contain theoretical relevance or conceptual insight into the phenomenon, allowing for a thorough examination of the research question.

#### 3.2.5 Dixon-Woods phase 5: Data extraction

Across the included CIS studies, data extraction approaches varied considerably, reflecting both structured and interpretive strategies. While many reviews reported using a data extraction table, spreadsheet, or pro forma (e.g., to organize descriptive information such as study aims, methodology, participants, and key findings), others engaged in more iterative and inductive processes consistent with the interpretive nature of CIS. Authors frequently described reading and re-reading included texts, identifying important or conceptually rich passages, and using coding, memo-ing, or mapping to document emergent insights. Some studies mentioned using tools to support extraction, such as a revised Critical Appraisal Tool (CAT) (*n* = 1), summary grids, feature maps, or categorization schemes tailored to conceptual constructs. Other studies described not using any formal extraction tool, instead engaging in dynamic synthesis processes where extraction, interpretation, and theme development occurred simultaneously. Software tools including NVivo (*n* = 6), Atlas.ti (*n* = 1), Covidence (*n* = 1), and the modified Dixon-Woods et al. data extraction tool (*n* = 2) were also used, particularly to support thematic coding and manage large volumes of data.

#### 3.2.6 Dixon-Woods phase 6: Interpretive synthesis

We evaluated the interpretive synthesis employed by the CIS reviews by examining how the synthesizing argument was generated, whether synthetic constructs or third-order constructs were generated, and whether the synthesizing argument included a critique of existing evidence. Synthetic constructs were generated by 15.6% (*n* = 33) of studies and 4.7% (*n* = 10) studies generated higher order constructs. The primary method of synthesizing the argument involved reading each article to iteratively determine where it fit into the larger body of literature. Subsequently, the articles were coded and transformed into themes, which served as a basis for the creation of synthetic constructs upon which the arguments were built. In 8.5% (*n* = 18) of studies, synthetic constructs and critique were integrated to generate a synthesizing argument.

Synthesis primarily involved constant comparison, critique, and exploration of themes across studies. Themes were consolidated into synthetic constructs, forming exploratory models. Reciprocal translation analysis, refutational synthesis, lines-of-argument synthesis, and taxonomy strategies were employed by several CIS. A critique of gaps and contradictions led to the identification of core concepts. Thematic categories were reconceptualized into three synthetic constructs, considering ontological and epistemological positions. Gaps in the data were discussed among research teams, and sometimes experts were consulted to refine the synthesized argument, which aimed to produce dynamic and nuanced understanding of the literature.

Some reviews performed and/or reported quality appraisal, data extraction, and data synthesis together. Quality appraisal and data extraction were both performed and reported together in 7.5% (*n* = 16) of reviews, whereas data extraction was both conducted and reported together with data synthesis in 44.8% (*n* = 95) of studies. One study combined quality appraisal, data extraction, and data synthesis, which were conducted and reported together. Many studies noted the recursive nature of the CIS and reported that different phases were interwoven or involved back-and-forth movement through the various stages, enabling authors to identify additional intersections of the data.

## 4 Discussion

The findings presented in this study illuminate the methodological landscape of CIS and provide valuable insights into its current state, trends, and challenges. Refining CIS methodology and its best practices are important for advancing evidence synthesis and knowledge generation across diverse disciplines and contexts. The analysis revealed a significant increase in the utilization of CIS methodology over the past 5 years, indicating a growing recognition of its value in synthesizing diverse bodies of literature within a wide and expanding range of fields. This upward trend suggests that researchers are increasingly turning to CIS to address complex research questions that require the integration of multiple perspectives and methodologies.

The primary motivations cited for employing CIS methodology revolve around its capacity to integrate data from heterogeneous sources and generate new insights and theoretical frameworks. CIS offers a flexible and iterative approach that allows researchers to navigate complex literature and develop nuanced interpretations. To maximize the effectiveness of CIS, researchers should clearly articulate the rationale for its use and align the methodology with the specific objectives of the study. Transparency and clarity in reporting the reasoning behind the choice of CIS methodology are essential for ensuring the credibility and trustworthiness of the findings.

However, conducting a CIS presents several challenges that researchers must address. These include the broad scope and diverse objectives of the existing literature, including gray literature, the variability in study designs and reporting quality, and the subjective nature of interpretive synthesis. The literature landscape included in a CIS requires researchers to navigate and integrate disparate perspectives and findings, reconcile inconsistent or incompatible forms of data, and manage the subjective nature of interpretive synthesis which adds another layer of complexity, as researchers must deal with individual interpretations and biases when synthesizing qualitative and mixed methods data. These challenges are further compounded by the lack of standardized methodological guidance.

The findings of this review highlight both the appeal and the complexity of conducting a CIS. While CIS is valued for its flexibility and capacity to integrate heterogeneous evidence, our analysis revealed substantial variation in how its methodological components are applied and reported. The greatest variability was observed in the phases of sampling and quality assessment, where only about half of the studies reported using formal strategies. In contrast, literature searching and data extraction were more consistently described, often using terminology and structures borrowed from systematic review methodology. This suggests that while CIS is intended to be a distinct and interpretive approach, in practice, many researchers continue to rely on familiar systematic review conventions, particularly in the early stages of the review process.

The Dixon-Woods et al.^1^ framework remains the most widely cited reference point, and studies mapped their processes to its six phases. However, few studies followed it in full, and many adapted or interpreted its components differently. For example, while the original framework emphasizes conceptual sampling based on theoretical relevance, many studies conflated this with systematic screening using predefined inclusion criteria. Similarly, quality assessment was inconsistently applied, with some studies omitting it entirely and others using a wide range of tools, often without justification. These inconsistencies reflect both the evolving nature of CIS and the lack of formal guidance on how to operationalize its principles.

While the Dixon-Woods framework provides a valuable foundation, our findings suggest that it serves as a flexible guide that researchers use to tailor to their specific contexts and aims. This flexibility is both a strength and a challenge as it allows CIS to be adapted to diverse research questions and disciplines, but it also complicates efforts to assess rigor, transparency, and comparability across studies. As CIS continues to gain traction, there is a growing need for minimum methodological expectations and reporting standards that preserve its interpretive richness while enhancing clarity.

Future research should focus on refining and formalizing the methodological components of CIS. This includes developing clearer guidance on conceptual sampling strategies, relevance assessment, and the integration of diverse data types. Comparative studies examining different approaches to conducting CIS could help identify best practices and inform the development of standardized reporting tools. Additionally, exploring how CIS can be adapted for emerging fields or interdisciplinary contexts may expand its utility. Finally, engaging with stakeholders and end-users of CIS findings could help ensure that methodological innovations align with the needs of policy, practice, and theory development.

### 4.1 Methodological elements

The iterative, inductive, and recursive nature of a CIS should be acknowledged and maintained in order to allow researchers to conduct thorough reviews. Building upon the methodological elements identified in previous research, researchers should adhere to the common practices for conducting a CIS by following an approach that encompasses the following steps (Table 1).

**Table 1:** Common practices for conducting a CIS

	Practical guidance based on most common practices
Dixon-Woods phase	identified in this review
Phase 1: Formulating the research question	Begin by clearly defining the research question or topic of inquiry, considering its relevance and significance to the field of study. Utilize subtopics and subquestions to further guide the study. Emphasize the exploratory and iterative nature of CIS, allowing for flexibility in refining the research question as the synthesis progresses.
Phase 2: Literature search	Conduct a comprehensive literature search using multiple sources, including electronic databases, specific key journals, reference chaining, websites of interest, and expert consultations. Employ systematic search strategies to identify relevant studies, including keywords, Boolean operators, and hand searching. Remove duplicates. Inclusion and exclusion criteria can be used for screening and to refine the search to identify potentially relevant studies for analysis. Additional searches can be done based on initial findings. Searches can be conducted, expanded, revised, and repeated.
Phase 3: Sampling	Use inclusion and exclusion criteria to select studies for analysis that are topically diverse and maximally relevant to research question or topic. Consider employing purposive, theoretical, or representative sampling methods to select studies based on their relevance and theoretical contribution and constructing a sampling frame.
	Prioritize degree of relevance over inclusion of all relevant literature, considering likelihood of record to contribute to the synthesis and generalizability of constructs. Ensure transparency in the sampling process, documenting the criteria used for inclusion and exclusion.
Phase 4: Quality assessment	Consider assessing the quality of included studies using standardized tools or frameworks, taking factors such as methodological rigor, validity, and reliability into consideration. Utilize quality assessment tools for peer-reviewed literature and gray literature. Quality assessment may be determined to be inappropriate for the study and may be skipped.
Phase 5. Data extraction	Systematically extract key data from included studies using a predefined data extraction form, template, or list of questions.
	Include primary data and interpretive data themes, frameworks, and conclusions. Employ rigorous methods for coding and thematic analysis, ensuring consistency and accuracy in the extraction process. Extraction and thematic codebook can be adjusted and adapted throughout to accommodate initial findings, concepts, and constructs.
Phase 6: Interpretive synthesis	Engage in interpretive synthesis by iteratively analyzing and synthesizing the findings of included studies. Generate synthetic constructs or higher order constructs to elucidate patterns, themes, and relationships within the literature, between studies, within groupings, and across the entire dataset. Key concepts and interpretations can be mapped against one another. Incorporate critical reflection and reflexivity throughout the synthesis process, acknowledging the subjective nature of interpretation. Engage authorship team, experts, and if relevant, stakeholders, throughout the synthesis process to enhance the interpretation, relevance, and validity of the findings. Synthesis should not be exclusively done as the final step of a process, rather taking into consideration throughout using tools such as memo-taking.

## 5 Strengths and limitations

The study conducted a thorough analysis of CIS methodology, encompassing a wide range of publications, and providing insights into trends, patterns, and practices. Employing a systematic approach for the scoping review and following established PRISMA guidelines for reporting ensures the reliability and reproducibility of the study’s findings. By including CIS reviews from various disciplines and geographical regions, the study provides a comprehensive overview of the application and adaptation of CIS methodology across different contexts. In addition, the study offers valuable guidance for researchers seeking to apply CIS methodology in their own studies. Despite the study’s strengths, the heterogeneity of included reviews may introduce variability in methodologies and practices, making it more challenging to draw generalizable conclusions or recommendations. The study also does not include a quality assessment of the included CIS reviews, and as such low-quality studies with poor methodology and reporting may have been included. Due to the large sample size of included articles, potential low-quality studies and different methodologies have been outweighed by conceptual saturation.

## 6 Conclusion

This study provides valuable insights into the methodological steps and highlights the most common practices for conducting a CIS. By understanding the rationale, limitations, and challenges associated with CIS, researchers can adopt strategies to optimize its utility and impact to ensure that the findings are relevant and actionable for informing policy, practice, and future research.

## Supporting information

Perlman et al. Supplementary Material 1 Perlman et al. supplementary material

Perlman et al. Supplementary Material 2 Perlman et al. supplementary material

## Data Availability

Data are available via the Open Science Framework (OSF) at https://osf.io/5trmh.

## Original publication

This work is an original unpublished work and has not been submitted.

## Supplementary material

To view supplementary material for this article, please visit http://doi.org/10.1017/rsm.2025.10041.
