# Supplementary material for: Making sense of conducting a critical interpretive synthesis: A scoping review
Source: Res Synth Methods. 2025 Oct 8;17(1):30–41. doi: 10.1017/rsm.2025.10041 (PMC12823206; doi:10.1017/rsm.2025.10041)
Supplement: Perlman et al. Supplementary Material 1 — Perlman et al. supplementary material [file S1759287925100410sup001.docx]

**Appendix 1:** Studies included in scoping review

| **Reference** | **Research context** | **Topic area** |
| --- | --- | --- |
| ^1^ Ako-Arrey DE, Brouwers MC, Lavis JN, Giacomini MK, AGREE-HS Team. Health systems guidance appraisal--a critical interpretive synthesis. *Implement Sci.* 2016;11:9. doi:10.1186/s13012-016-0373-y | Health- Healthcare delivery, policy and research | health systems guidance |
| ^2^ Alam B, Kaler A, Mumtaz Z. Women’s voices and medical abortions: A review of the literature. *Eur J Obstet Gynecol Reprod Biol.* 2020;249:21-31. doi:10.1016/j.ejogrb.2020.04.003 | Health- Women's and maternal health | women's perspectives of medical abortion |
| ^3^ Allen B, Bond C. The educational psychologist’s role in child protection and safeguarding: an exploration of research over time. *Educational Psychology in Practice.* 2020;36(4):386-404. doi:10.1080/02667363.2020.1809353 | Psychology | educational psychologist's role in child protection and safeguarding |
| ^4^ Al Sabahi S, Wilson MG, Lavis JN, El-Jardali F, Moat K, Vélez M. Examining and Contextualizing Approaches to Establish Policy Support Organizations - A Critical Interpretive Synthesis. *Int J Health Policy Manag.* 2022;11(5):551-566. doi:10.34172/ijhpm.2020.181 | Knowledge translation | evidence-informed policymaking/policy support organizations |
| ^5^ Alvarez E. Developing and evaluating the use of a workbook forcontextualizing health systems guidance [Doctoral dissertation]. 2016. | Health- Healthcare delivery, policy and research | contextualization of guidance recommendations in health systems |
| ^6^ Andrews S, Hamilton B, Humphreys C. A global silence: A critical interpretive synthesis of aboriginal mothering through domestic and family violence. *Affilia.* 2022;37(4):585-604. doi:10.1177/08861099211055520 | Social work | domestic and family violence against aboriginal women |
| ^7^ Annandale E, Harvey J, Cavers D, Dixon-Woods M. Gender and access to healthcare in the UK: a critical interpretive synthesis of the literature. *Evidence & Policy: A Journal of Research, Debate and Practice.* 2007;3(4):463-486. doi:10.1332/174426407782516538 | Health- Health equity and public health | gender and healthcare access |
| ^8^ Balaam M-C, Kingdon C, Haith-Cooper M. A Systematic Review of Perinatal Social Support Interventions for Asylum-seeking and Refugee Women Residing in Europe. *J Immigr Minor Health*. 2022;24(3):741-758. doi:10.1007/s10903-021-01242-3 | Health- Health equity and public health | perinatal social support interventions for the wellbeing of asylum-seeking and refugee women in the EU |
| ^9^ Bales S, Gee C. Critical interpretive synthesis for informing collection decisions. *Collection Building.* 2012;32(2):51–6. | Journalism | library selector decision making |
| ^10^ Ball E, McLoughlin M, Darvill A. Plethora or paucity: a systematic search and bibliometric study of the application and design of qualitative methods in nursing research 2008-2010. *Nurse Educ Today.* 2011;31(3):299–303. doi:10.1016/j.nedt.2010.12.002 | Health- Nursing and midwifery | design, use, application and rigour of qualitative methodology in nursing research |
| ^11^ Bargeman M, Abelson J, Mulvale G, Niec A, Theuer A, Moll S. Understanding the Conceptualization and Operationalization of Trauma-Informed Care Within and Across Systems: A Critical Interpretive Synthesis. *Milbank Q.* 2022;100(3):785–853. doi:10.1111/1468-0009.12579 | Trauma | trauma-informed care |
| ^12^ Beňová L, Semaan A, Portela A, Bonet M, van den Akker T, Pembe AB, et al. Facilitators and barriers of implementation of routine postnatal care guidelines for women: A systematic scoping review using critical interpretive synthesis. *J Glob Health.* 2023;13:04176. doi:10.7189/jogh.13.04176 | Health- Women's and maternal health | post-natal care |
| ^13^ Benjamin-Thomas TE, Rudman DL. A critical interpretive synthesis: Use of the occupational justice framework in research. *Aust Occup Ther J*. 2018;65(1):3–14. doi:10.1111/1440-1630.12428 | Occupational justice | extent of the use of the occupational justice framework within research |
| ^14^ Bibb J, Baker FA, Skewes McFerran K. A critical interpretive synthesis of the most commonly used self-report measures in Australian mental health research. *Australas Psychiatry*. 2016;24(5):453-458. doi:10.1177/1039856215626646 | Health- Mental health | self-report measures in mental health research |
| ^15^ Borst RAJ, Wehrens R, Bal R. Sustaining knowledge translation practices: A critical interpretive synthesis. *Int J Health Policy Manag*. 2022;11(12):2793-2804. doi:10.34172/ijhpm.2022.6424 | Knowledge translation | sustaining KT practices |
| ^16^ Bourret K, Mattison C, Hebert E, et al. Evidence-informed framework for gender transformative continuing education interventions for midwives and midwifery associations. *BMJ Glob Health*. 2023;8(1). doi:10.1136/bmjgh-2022-011242 | Health- Nursing and midwifery | continuing education interventions for midwives |
| ^17^ Boutcher F, Berta W, Urquhart R, Gagliardi AR. The roles, activities and impacts of middle managers who function as knowledge brokers to improve care delivery and outcomes in healthcare organizations: a critical interpretive synthesis. *BMC Health Serv Res*. 2022;22(1):11. doi:10.1186/s12913-021-07387-z | Knowledge translation | middle managers as knowledge brokers |
| ^18^ Boyko JA, Lavis JN, Abelson J, Dobbins M, Carter N. Deliberative dialogues as a mechanism for knowledge translation and exchange in health systems decision-making. *Soc Sci Med*. 2012;75(11):1938-1945. doi:10.1016/j.socscimed.2012.06.016 | Knowledge translation | deliberate dialogues as a KTE strategy and evidence-informed policymaking support tool |
| ^19^ Brownie SM. Nurses in Health Service Leadership: The Power to Influence [Doctoral dissertation]. 2021. | Health- Nursing and midwifery | enhancing nurses' effective policy engagement in global health systems |
| ^20^ Bullock HL, Lavis JN, Wilson MG, Mulvale G, Miatello A. Understanding the implementation of evidence-informed policies and practices from a policy perspective: a critical interpretive synthesis. *Implement Sci*. 2021;16(1):18. doi:10.1186/s13012-021-01082-7 | Implementation science | implementation process from an integrated policy perspective |
| ^21^ Cameron JE. Rituals surrounding the care of the dying previable baby in labour ward: a critical interpretive synthesis of the literature. [Doctoral dissertation]. 2011. | Health- Nursing and midwifery | rituals surrounding the care of the dying previable baby |
| ^22^ Carboni C, Wehrens R, van der Veen R, de Bont A. Conceptualizing the digitalization of healthcare work: A metaphor-based Critical Interpretive Synthesis. *Soc Sci Med*. 2022;292:114572. doi:10.1016/j.socscimed.2021.114572 | Health- Healthcare delivery, policy and research | digitalization of healthcare work |
| ^23^ Carlsson I-M, Larsson I, Jormfeldt H. Place and space in relation to childbirth: a critical interpretive synthesis. *Int J Qual Stud Health Well-being*. 2020;15(sup1):1667143. doi:10.1080/17482631.2019.1667143 | Health- Nursing and midwifery | concept of place and space in relation to childbirth |
| ^24^ Chalmiers MA, Karaki F, Muriki M, Mody SK, Chen A, Thiel de Bocanegra H. Refugee women’s experiences with contraceptive care after resettlement in high-income countries: A critical interpretive synthesis. *Contraception*. 2022;108:7-18. doi:10.1016/j.contraception.2021.11.004 | Health- Health equity and public health | experiences with contraceptive care after resettlement in high-income countries |
| ^25^ Chambers D, Cantrell A, Sworn K, Booth A. *Assessment and Management Pathways of Older Adults with Mild Cognitive Impairment: Descriptive Review and Critical Interpretive Synthesis*. Southampton (UK): National Institute for Health and Care Research; 2022. doi:10.3310/XLUJ6074 | Health- Clinical health services | mild cognitive impairment |
| ^26^ Clark MT, Vakaj E, Biernat KN, McKnight LK, Cowdell F. Knowledge mobilisation in safeguarding adults and children for healthcare in England. *Health Soc Care Community*. 2023;2023:1-25. doi:10.1155/2023/6080695 | Knowledge translation | safeguarding for healthcare |
| ^27^ Clark MT, Clark RJ, Toohey S, Bradbury-Jones C. Rationales and treatment approaches underpinning the use of acupuncture and related techniques for plantar heel pain: a critical interpretive synthesis. *Acupunct Med*. 2017;35(1):9-16. doi:10.1136/acupmed-2015-011042 | Health- Clinical health services | acupuncture for plantar heel pain |
| ^28^ Clark J, Gnanapragasam S, Greenley S, Pearce J, Johnson M. Perceptions and experiences of laws and regulations governing access to opioids in South, Southeast, East and Central Asia: A systematic review, critical interpretative synthesis and development of a conceptual framework. *Palliat Med*. 2021;35(1):59-75. doi:10.1177/0269216320966505 | Health- Health equity and public health | opioid access |
| ^29^ Comte R. Neo-colonialism In Music Therapy: A Critical Interpretive Synthesis of the Literature Concerning Music Therapy Practice With Refugees. *Voices*. 2016;16(3). doi:10.15845/voices.v16i3.865 | Music therapy | music therapy with refugees |
| ^30^ Conley Wright A, Metcalfe L, Heward-Belle S, Collings S, Barrett E. *Critical Interpretive Synthesis: Child Protection Involvement for Families with Domestic and Family Violence, Alcohol and Other Drug Issues, and Mental Health Issues.* Australia’s National Research Organisation for Women’s Safety (ANROWS); 2021. | Health- Mental health | child protection |
| ^31^ Cooke J, Moodley P. Indicators for continuance of childhood gender dysphoria into adulthood: A critical interpretive synthesis of literature (2000–2020). *Int Health Trends & Persp*. 2023;3(1):44-70. doi:10.32920/ihtp.v3i1.1690 | Psychology | childhood gender dysphoria |
| ^32^ Corrado AM, Benjamin-Thomas TE, McGrath C, Hand C, Laliberte Rudman D. Participatory action research with older adults: A critical interpretive synthesis. *Gerontologist*. 2020;60(5):e413-e427. doi:10.1093/geront/gnz080 | Public policy | older adults' participatory action research |
| ^33^ Cox CL, Miller BM, Kuhn I, Fritz Z. Diagnostic uncertainty in primary care: what is known about its communication, and what are the associated ethical issues? *Fam Pract*. 2021;38(5):654-668. doi:10.1093/fampra/cmab023 | Health- Clinical health services | diagnostic uncertainty in primary care |
| ^34^ Daker-White G, Rogers A. What is the potential for social networks and support to enhance future telehealth interventions for people with a diagnosis of schizophrenia: a critical interpretive synthesis. *BMC Psychiatry*. 2013;13:279. doi:10.1186/1471-244X-13-279 | Health- Healthcare delivery, policy and research | telehealth interventions, social networks and peer support for people with a diagnosis of schizophrenia |
| ^35^ Daniel S, Clark J, Gnanapragasam S, Venkateswaran C, Johnson MJ. Psychological concerns of Indian women with breast cancer in different national contexts: a systematic review and mixed-methods synthesis. *BMJ Support Palliat Care*. 2022;12(e4):e537-e549. doi:10.1136/bmjspcare-2019-002076 | Health- Clinical health services | psychological symptoms of women with breast cancer |
| ^36^ Dash MJ, Hamdani Y, Laliberte Rudman D, Teachman G. Representations of parenting autistic children: A critical interpretive synthesis. *Scand J Occup Ther*. 2023;30(8):1209-1223. doi:10.1080/11038128.2023.2210802 | Occupational therapy | parenting autistic children |
| ^37^ Davis LF, Ramírez-Andreotta MD. Participatory research for environmental justice: A critical interpretive synthesis. *Environ Health Perspect*. 2021;129(2):26001. doi:10.1289/EHP6274 | Health- Health equity and public health | prompting structural change in environmental justice communities |
| ^38^ de Bie A. Teaching with madness/’mental illness’ autobiographies in postsecondary education: ethical and epistemological implications. *Med Humanit*. 2022;48(1):37-50. doi:10.1136/medhum-2020-011974 | Higher education | pedagogical use of madness in postsecondary education |
| ^39^ Deering K, Brimblecombe N, Matonhodze JC, Nolan F, Collins DA, Renwick L. Methodological procedures for priority setting mental health research: a systematic review summarising the methods, designs and frameworks involved with priority setting. *Health Res Policy Syst*. 2023;21(1):64. doi:10.1186/s12961-023-01003-8 | Health- Mental health | methodological procedures for priority setting mental health research |
| ^40^ DeFeo NJ. How culture informs hospice music therapy: a critical interpretive synthesis [Doctoral dissertation]. 2017. | Music therapy | hospice music therapy |
| ^41^ Degrie L, Gastmans C, Mahieu L, Dierckx de Casterlé B, Denier Y. “How do ethnic minority patients experience the intercultural care encounter in hospitals? a systematic review of qualitative research”. *BMC Med Ethics*. 2017;18(1):2. doi:10.1186/s12910-016-0163-8 | Health- Health equity and public health | intercultural care encounters in hospitals |
| ^42^ Denburg AE, Giacomini M, Ungar WJ, Abelson J. The moral foundations of child health and social policies: A critical interpretive synthesis. *Children (Basel)*. 2021;8(1). doi:10.3390/children8010043 | Health- Healthcare delivery, policy and research | normative dimensions of child health and social policymaking |
| ^43^ Dixon-Woods M, Cavers D, Agarwal S, et al. Conducting a critical interpretive synthesis of the literature on access to healthcare by vulnerable groups. *BMC Med Res Methodol*. 2006;6:35. doi:10.1186/1471-2288-6-35 | Health- Healthcare delivery, policy and research | access to healthcare |
| ^44^ Duan Y, Iaconi A, Wang J, et al. Conceptual and relational advances of the PARIHS and i-PARIHS frameworks over the last decade: a critical interpretive synthesis. *Implement Sci*. 2022;17(1):78. doi:10.1186/s13012-022-01254-z | Implementation science | (i-)PARIHS framework |
| ^45^ Du Toit SHJ, Shen X, McGrath M. Meaningful engagement and person-centered residential dementia care: A critical interpretive synthesis. *Scand J Occup Ther*. 2019;26(5):343-355. doi:10.1080/11038128.2018.1441323 | Occupational therapy | residential dementia care |
| ^46^ Edwards J. The extant rationale for mandated therapy during psychotherapy and counselling training: a critical interpretive synthesis. *Br J Guid Counc*. 2018;46(5):515-530. doi:10.1080/03069885.2017.1334110 | Health- Mental health | mandated therapy |
| ^47^ Edwards J. Counseling and psychology student experiences of personal therapy: A critical interpretive synthesis. *Front Psychol*. 2018;9:1732. doi:10.3389/fpsyg.2018.01732 | Health- Mental health | personal therapy for trainee therapists |
| ^48^ Ellen ME, Wilson MG, Vélez M, et al. Addressing overuse of health services in health systems: a critical interpretive synthesis. *Health Res Policy Syst*. 2018;16(1):48. doi:10.1186/s12961-018-0325-x | Health- Healthcare delivery, policy and research | overuse of health services |
| ^49^ Engel L, Bryan S, Whitehurst DGT. Conceptualising “Benefits Beyond Health” in the Context of the Quality-Adjusted Life-Year: A Critical Interpretive Synthesis. *Pharmacoeconomics*. 2021;39(12):1383-1395. doi:10.1007/s40273-021-01074-x | Health- Clinical health services | Quality-Adjusted Life-Year |
| ^50^ Entwistle V, Firnigl D, Ryan M, Francis J, Kinghorn P. Which experiences of health care delivery matter to service users and why? A critical interpretive synthesis and conceptual map. *J Health Serv Res Policy*. 2012;17(2):70-78. doi:10.1258/jhsrp.2011.011029 | Health- Healthcare delivery, policy and research | healthcare delivery experience |
| ^51^ Eskyte I, Manzano A, Pepper G, et al. Understanding treatment decisions from the perspective of people with relapsing remitting multiple Sclerosis: A critical interpretive synthesis. *Mult Scler Relat Disord*. 2019;27:370-377. doi:10.1016/j.msard.2018.11.016 | Health- Clinical health services | multiple sclerosis treatment decisions |
| ^52^ Essex R, Weldon SM. The justification for strike action in healthcare: A systematic critical interpretive synthesis. *Nurs Ethics*. 2022;29(5):1152-1173. doi:10.1177/09697330211022411 | Health- Health equity and public health | strike action in healthcare |
| ^53^ Evans C, Abelson J, Kates N, Cavanagh A, Lavis JN. A multilevel framework for complex care: A critical interpretive synthesis. *Health Soc Care Community*. 2023;2023:1-14. doi:10.1155/2023/4487200 | Health- Clinical health services | complex care policy |
| ^54^ Fairchild R, McFerran KS, Thompson G. A critical interpretive synthesis of the ways children’s needs and capacities are represented in the homelessness and family violence literature. *Children Australia*. 2017;42(1):18-29. doi:10.1017/cha.2016.46 | Welfare | children's needs within homelessness and family violence |
| ^55^ Fane J, MacDougall C, Redmond G, Jovanovic J, Ward P. Young children’s health and wellbeing across the transition to school: A critical interpretive synthesis. *Children Australia*. 2016;41(2):126-140. doi:10.1017/cha.2016.4 | Health- Health equity and public health | transition to school for young children |
| ^56^ Farias L, Rudman DL. A critical interpretive synthesis of the uptake of critical perspectives in occupational science. *Journal of Occupational Science*. 2016;23(1):33-50. doi:10.1080/14427591.2014.989893 | Occupational science | uptake of critical perspectives |
| ^57^ Farrelly S, Lester H. Therapeutic relationships between mental health service users with psychotic disorders and their clinicians: a critical interpretive synthesis. *Health Soc Care Community*. 2014;22(5):449-460. doi:10.1111/hsc.12090 | Health- Mental health | therapeutic relationships between service users and clinicians |
| ^58^ Fish J. How does assisted dying affect the experience of the bereaved in the UK? [Doctoral dissertation]. 2023. | Suicide | experiences of caregivers who supported a patient through assisted suicide |
| ^59^ Flemming K. The use of morphine to treat cancer-related pain: a synthesis of quantitative and qualitative research. *J Pain Symptom Manage*. 2010;39(1):139-154. doi:10.1016/j.jpainsymman.2009.05.014 | Health- Clinical health services | cancer-related pain |
| ^60^ Flemming K. Synthesis of quantitative and qualitative research: an example using Critical Interpretive Synthesis. *J Adv Nurs*. 2010;66(1):201-217. doi:10.1111/j.1365-2648.2009.05173.x | Research methodology | synthesis of quantitative and qualitative research |
| ^61^ Forde H, Chavez-Ugalde Y, Jones RA, et al. The conceptualisation and operationalisation of “marketing” in public health research: a review of reviews focused on food marketing using principles from critical interpretive synthesis. *BMC Public Health*. 2023;23(1):1419. doi:10.1186/s12889-023-16293-4 | Health- Health equity and public health | marketing in public health research of food marketing |
| ^62^ Foroughi Z, Ebrahimi P, Aryankhesal A, Maleki M, Yazdani S. Toward a theory-led meta-framework for implementing health system resilience analysis studies: a systematic review and critical interpretive synthesis. *BMC Public Health*. 2022;22(1):287. doi:10.1186/s12889-022-12496-3 | Health- Healthcare delivery, policy and research | health system resilience |
| ^63^ French C, Dowrick A, Fudge N, Pinnock H, Taylor SJC. What do we want to get out of this? a critical interpretive synthesis of the value of process evaluations, with a practical planning framework. *BMC Med Res Methodol*. 2022;22(1):302. doi:10.1186/s12874-022-01767-7 | Health- Healthcare delivery, policy and research | process evaluation |
| ^64^ Göransson C, Larsson I, Carlsson I-M. Art of connectedness: Value-creating care for older persons provided with toileting assistance and containment strategies-A critical interpretive synthesis. *J Clin Nurs*. 2023;32(9-10):1806-1820. doi:10.1111/jocn.16216 | Health- Clinical health services | value-creating care for older adults |
| ^65^ Gambe RG, Clark J, Meddick-Dyson SA, Ukoha-Kalu BO, Nyaaba GN, Murtagh FEM. The roles and experiences of informal carers providing care to people with advanced cancer in Africa-A systematic review and critical interpretive analysis. *PLOS Glob Public Health*. 2023;3(4):e0001785. doi:10.1371/journal.pgph.0001785 | Health- Clinical health services | roles and experiences of informal carers |
| ^66^ Garrido S, Dunne L, Chang E, Perz J, Stevens CJ, Haertsch M. The Use of Music Playlists for People with Dementia: A Critical Synthesis. *J Alzheimers Dis*. 2017;60(3):1129-1142. doi:10.3233/JAD-170612 | Music therapy | use of music for dementia |
| ^67^ Georgiou N, Morgan RM, French JC. Conceptualising, evaluating and communicating uncertainty in forensic science: Identifying commonly used tools through an interdisciplinary configurative review. *Sci Justice*. 2020;60(4):313-336. doi:10.1016/j.scijus.2020.04.002 | Forensic science | uncertainty in forensic science |
| ^68^ Ghinea N, Wiersma M, Newson AJ, Walby C, Norman RJ, Lipworth W. Situating commercialization of assisted reproduction in its socio-political context: a critical interpretive synthesis. *Hum Reprod Open*. 2022;2022(4):hoac052. doi:10.1093/hropen/hoac052 | Health- Women's and maternal health | commercialization of assisted reproduction |
| ^69^ Gullmark P, Clausen TH. In search of innovation capability and its sources in local government organizations: a critical interpretative synthesis of the literature. *International Public Management Journal*. 2023;26(2):258-280. doi:10.1080/10967494.2022.2157917 | Public sector | innovation local government organization |
| ^70^ Gysels MH, Evans C, Higginson IJ. Patient, caregiver, health professional and researcher views and experiences of participating in research at the end of life: a critical interpretive synthesis of the literature. *BMC Med Res Methodol*. 2012;12:123. doi:10.1186/1471-2288-12-123 | Health- Clinical health services | end-of-life patient involvement |
| ^71^ Haby MM, Chapman E, Barreto JOM, et al. Greater agreement is required to harness the potential of health intelligence: a critical interpretive synthesis. *J Clin Epidemiol*. 2023;163:37-50. doi:10.1016/j.jclinepi.2023.09.007 | Health- Healthcare delivery, policy and research | health intelligence |
| ^72^ Haddrill R, Jones GL, Anumba D, Mitchell C. A tale of two pregnancies: A Critical Interpretive Synthesis of women’s perceptions about delayed initiation of antenatal care. *Women Birth*. 2018;31(3):220-231. doi:10.1016/j.wombi.2017.09.017 | Health- Women's and maternal health | women's perceptions regarding antenatal care |
| ^73^ Hagedorn CA. Towards improved intra-organizational information sharing: The intelligent community college’s contact center [Doctoral dissertation]. 2018. | Higher education | use of college knowledge and information sharing and management |
| ^74^ Harris RV, Pennington A, Whitehead M. Preventive dental visiting: a critical interpretive synthesis of theory explaining how inequalities arise. *Community Dent Oral Epidemiol*. 2017;45(2):120-134. doi:10.1111/cdoe.12268 | Dentistry | inequality in dental care |
| ^75^ Harrison SL, Apps L, Singh SJ, Steiner MC, Morgan MDL, Robertson N. “Consumed by breathing” - a critical interpretive meta-synthesis of the qualitative literature. *Chronic Illn*. 2014;10(1):31-49. doi:10.1177/1742395313493122 | Health- Clinical health services | patient experiences of chronic obstructive pulmonary disease |
| ^76^ Harrison M, Rhodes T, Lancaster K. How do care environments shape healthcare? A synthesis of qualitative studies among healthcare workers during the COVID-19 pandemic. *BMJ Open*. 2022;12(9):e063867. doi:10.1136/bmjopen-2022-063867 | Health- Healthcare delivery, policy and research | shaping care environments |
| ^77^ Haugen I, Slettebø T, Ytrehus S. Factors affecting user participation for elderly people with dementia living at home: a critical interpretive synthesis of the literature. *European Journal of Social Work*. 2019;22(6):974-986. doi:10.1080/13691457.2018.1441133 | Social work | user participation dementia patients living at home |
| ^78^ Heaton J, Corden A, Parker G. “Continuity of care”: a critical interpretive synthesis of how the concept was elaborated by a national research programme. *Int J Integr Care*. 2012;12:e12. doi:10.5334/ijic.794 | Health- Clinical health services | continuity of care |
| ^79^ Heggernes SL. A critical review of the role of texts in fostering Intercultural Communicative competence in the English Language classroom. *Educational Research Review*. 2021;33:100390. doi:10.1016/j.edurev.2021.100390 | Education | intercultural learning in English |
| ^80^ Hock R, Mooradian J. Defining Coparenting for Social Work Practice: A Critical Interpretive Synthesis. *Journal of Family Social Work*. 2013;16(4):314-331. | Social work | coparenting |
| ^81^ Hodge G. Dementia and its relationship with suicidality: A critical interpretive synthesis. *Dementia*. 2020;19(5):1397-1412. doi:10.1177/1471301218799871 | Health- Clinical health services | dementia and suicide |
| ^82^ Holmes MM, Lewith G, Newell D, Field J, Bishop FL. The impact of patient-reported outcome measures in clinical practice for pain: a systematic review. *Qual Life Res*. 2017;26(2):245-257. doi:10.1007/s11136-016-1449-5 | Health- Clinical health services | patient-reported outcome measures for pain |
| ^83^ House J, Kleiber D, Steenbergen DJ, Stacey N. Participatory monitoring in community-based fisheries management through a gender lens. *Ambio*. 2023;52(2):300-318. doi:10.1007/s13280-022-01783-3 | Fishery management | gender or participatory monitoring |
| ^84^ Howes S, Warwick P. Creating equitable and sustainable opportunities for nature immersion to support restoration from stress within mental health nursing: A critical interpretive synthesis. *Int J Ment Health Nurs*. 2023;32(3):673-686. doi:10.1111/inm.13109 | Health- Mental health | mental health nursing |
| ^85^ Huber C, Montreuil C, Christie D, Forbes A. Integrating Self-Management Education and Support in Routine Care of People With Type 2 Diabetes Mellitus: A Conceptional Model Based on Critical Interpretive Synthesis and A Consensus-Building Participatory Consultation. *Front Clin Diabetes Healthc*. 2022;3. doi:10.3389/fcdhc.2022.845547 | Health- Clinical health services | diabetes self-management |
| ^86^ Hudon A, Lippel K, MacEachen E. Mapping first-line health care providers’ roles, practices, and impacts on care for workers with compensable musculoskeletal disorders in four jurisdictions: A critical interpretive synthesis. *Am J Ind Med*. 2019;62(7):545-558. doi:10.1002/ajim.22972 | Health- Clinical health services | health care providers' roles, practices, and impacts |
| ^87^ Hudson KD, Mehrotra GR. Intersectional Social Work Practice: A Critical Interpretive Synthesis of Peer-Reviewed Recommendations. *Families in Society: The Journal of Contemporary Social Services*. 2021;102(2):140-153. doi:10.1177/1044389420964150 | Social work | intersectionality in social work |
| ^88^ Ingersoll JS. Factors to Consider When Balancing Campus Safety Concerns with Students’ Civil Rights [Doctoral dissertation]. 2017. | Civil rights | campus safety and civil rights |
| ^89^ Iriarte-Roteta A, Lopez-Dicastillo O, Mujika A, et al. Nurses’ role in health promotion and prevention: A critical interpretive synthesis. *J Clin Nurs*. 2020;29(21-22):3937-3949. doi:10.1111/jocn.15441 | Health- Nursing and midwifery | role of nurses for health promotion and prevention |
| ^90^ Isobe J, Healey L, Humphreys C. A critical interpretive synthesis of the intersection of domestic violence with parental issues of mental health and substance misuse. *Health Soc Care Community*. 2020;28(5):1394-1407. doi:10.1111/hsc.12978 | Social work | intersection of domestic violence and mental health |
| ^91^ Isobel S, Goodyear M, Furness T, Foster K. Preventing intergenerational trauma transmission: A critical interpretive synthesis. *J Clin Nurs*. 2019;28(7-8):1100-1113. doi:10.1111/jocn.14735 | Trauma | intergenerational trauma transmission |
| ^92^ Jakubowski BE, Hinton L, Khaira J, Roberts N, McManus RJ, Tucker KL. Is self-management a burden? What are the experiences of women self-managing chronic conditions during pregnancy? A systematic review. *BMJ Open*. 2022;12(3):e051962. doi:10.1136/bmjopen-2021-051962 | Health- Women's and maternal health | self-managing chronic conditions during pregnancy |
| ^93^ Jansen van Rensburg N, Spies R, Malan L. Infanticide and its relationship with postpartum psychosis: a critical interpretive synthesis. *Jcp*. 2020;10(4):293-310. doi:10.1108/JCP-05-2020-0018 | Health- Women's and maternal health | infanticide and postpartum psychosis |
| ^94^ Jaramillo-Yanquepe C. A synthesis of EFL research in chilean high schools: research shortage or research opportunities? *Profile: Issues Teach Prof Dev*. 2022;24(1):227-246. doi:10.15446/profile.v24n1.92155 | Education | English as a foreign language education |
| ^95^ Jarvis T, Scott F, El-Jardali F, Alvarez E. Defining and classifying public health systems: a critical interpretive synthesis. *Health Res Policy Syst*. 2020;18(1):68. doi:10.1186/s12961-020-00583-z | Health- Healthcare delivery, policy and research | public health systems |
| ^96^ Johnson M, Tod AM, Brummell S, Collins K. Prognostic communication in cancer: A critical interpretive synthesis of the literature. *Eur J Oncol Nurs*. 2015;19(5):554-567. doi:10.1016/j.ejon.2015.03.001 | Health- Clinical health services | prognostic communication |
| ^97^ Kangasniemi M, Kallio H, Pietilä A-M. Towards environmentally responsible nursing: a critical interpretive synthesis. *J Adv Nurs*. 2014;70(7):1465-1478. doi:10.1111/jan.12347 | Health- Nursing and midwifery | environmental issues in nursing |
| ^98^ Kazimierczak KA, Skea ZC, Dixon-Woods M, et al. Provision of cancer information as a “support for navigating the knowledge landscape”: findings from a critical interpretive literature synthesis. *Eur J Oncol Nurs*. 2013;17(3):360-369. doi:10.1016/j.ejon.2012.10.002 | Health- Clinical health services | cancer patient engagement |
| ^99^ Kekkonen M, Böök ML, Kokkinen K, et al. An interpretative synthesis of coparenting among new parents in diverse sociocultural contexts. *J Comp Fam Stud*. 2023;54(1):6-29. doi:10.3138/jcfs.54.1.030 | Parenting | co-parenting in cultural diversity |
| ^100^ Kelly CA, Maden M. How do health-care professionals perceive oxygen therapy? A critical interpretive synthesis of the literature. *Chron Respir Dis*. 2015;12(1):11-23. doi:10.1177/1479972314562408 | Health- Clinical health services | oxygen therapy |
| ^101^ Keygnaert I, Guieu A, Ooms G, Vettenburg N, Temmerman M, Roelens K. Sexual and reproductive health of migrants: does the EU care? *Health Policy*. 2014;114(2-3):215-225. doi:10.1016/j.healthpol.2013.10.007 | Health- Health equity and public health | migrant sexual health |
| ^102^ Khalid AF, Lavis JN, El-Jardali F, Vanstone M. Supporting the use of research evidence in decision-making in crisis zones in low- and middle-income countries: a critical interpretive synthesis. *Health Res Policy Syst*. 2020;18(1):21. doi:10.1186/s12961-020-0530-2 | Health- Healthcare delivery, policy and research | use of research evidence in decision-making |
| ^103^ Kim SY. The experiences of adults with autism spectrum disorder: Self-determination and quality of life. *Res Autism Spectr Disord*. 2019;60:1-15. doi:10.1016/j.rasd.2018.12.002 | Health- Health equity and public health | quality of life for adults with autism spectrum disorder |
| ^104^.Kimport K, Littlejohn KE. What are we forgetting? sexuality, sex, and embodiment in abortion research. *J Sex Res*. 2021;58(7):863-873. doi:10.1080/00224499.2021.1925620 | Social policy | sexuality and abortion |
| ^105^ Kuchenmüller T, Boeira L, Oliver S, et al. Domains and processes for institutionalizing evidence-informed health policy-making: a critical interpretive synthesis. *Health Res Policy Syst*. 2022;20(1):27. doi:10.1186/s12961-022-00820-7 | Health- Healthcare delivery, policy and research | institutionalizing evidence-informed health policy-making |
| ^106^ Kurup C, Burston A, Miles S. Transition of internationally qualified nurses in Australia: Meta-synthesis of qualitative studies. *Collegian*. 2023;30(2):357-366. doi:10.1016/j.colegn.2022.10.002 | Health- Healthcare delivery, policy and research | transitioning of internationally qualified nurses |
| ^107^ Laff RH. A global perspective on mindfulness-based interventions in schools [Doctoral dissertation]. 2023. | Education | mindfulness-based interventions utilized in classroom settings |
| ^108^ Lakin K, Kane S. A critical interpretive synthesis of migrants’ experiences of the Australian health system. *Int J Equity Health*. 2023;22(1):7. doi:10.1186/s12939-022-01821-2 | Health- Healthcare delivery, policy and research | migrant experiences health system |
| ^109^ Laliberte Rudman D, Egan MY, McGrath CE, et al. Low Vision Rehabilitation, Age-Related Vision Loss, and Risk: A Critical Interpretive Synthesis. *Gerontologist*. 2016;56(3):e32-45. doi:10.1093/geront/gnv685 | Health- Clinical health services | rehab for low vision |
| ^110^ Lambert E, Strickland K, Gibson J. Cultural considerations at end-of-life for people of culturally and linguistically diverse backgrounds: A critical interpretative synthesis. *J Clin Nurs*. April 2023. doi:10.1111/jocn.16710 | Health- Clinical health services | cultural considerations at end-of-life |
| ^111^ Lamsal R. Measuring and incorporating family spillover cost and health consequences in economic evaluation of child health interventions. [Doctoral dissertation]. 2023. | Health- Healthcare delivery, policy and research | incorporating family spillover effects in pediatric economic evaluations |
| ^112^ Law JH, Sultan N, Finer S, Fudge N. Advancing the communication of genetic risk for cardiometabolic diseases: a critical interpretive synthesis. *BMC Med*. 2023;21(1):432. doi:10.1186/s12916-023-03150-9 | Health- Clinical health services | communication of genetic risk for cardiometabolic diseases |
| ^113^ Lawrence DS, Ssali A, Jarvis JN, Seeley J. Clinical research for life-threatening illnesses requiring emergency hospitalisation: a critical interpretive synthesis of qualitative data related to the experience of participants and their caregivers. *Trials*. 2023;24(1):149. doi:10.1186/s13063-023-07183-6 | Health- Healthcare delivery, policy and research | design clinical trials for life-threatening illnesses |
| ^114^ Lees A, Ryan AM, Muñoz M, Tocci C. Mapping the indigenous postcolonial possibilities of teacher preparation. *Journal of Teacher Education*. September 2023. doi:10.1177/00224871231199361 | Education | teacher education programs for decolonization |
| ^115^ Legg R, Prior J, Adams J, McIntyre E. The relations between mental health and psychological wellbeing and living with environmental contamination: A systematic review and conceptual framework. *J Environ Psychol*. 2023;87:101994. doi:10.1016/j.jenvp.2023.101994 | Health- Mental health | mental health and psychological wellbeing in environmental contamination experience |
| ^116^ Lemchak B. Music therapy at the end of life: a critical interpretive synthesis of the literature [Doctoral dissertation]. 2017. | Music therapy | music therapy at end-of-life |
| ^117^ Lin S, Melendez-Torres GJ. Critical interpretive synthesis of barriers and facilitators to TB treatment in immigrant populations. *Trop Med Int Health*. 2017;22(10):1206-1222. doi:10.1111/tmi.12938 | Health- Health equity and public health | tuberculosis treatment in immigrants |
| ^118^ Lippmann M, Laudel H, Heinzle M, Narciss S. Relating Instructional Design Components to the Effectiveness of Internet-Based Mindfulness Interventions: A Critical Interpretive Synthesis. *J Med Internet Res*. 2019;21(11):e12497. doi:10.2196/12497 | Mindfullness | internet-based mindfulness interventions |
| ^119^ Luddy J. The Ritual Lens: Student Success in Community Colleges [Doctoral dissertation]. 2017. | Higher education | student success in community colleges |
| ^120^ Mackenzie M, Conway E, Hastings A, Munro M, O’Donnell C. Is ‘Candidacy’ a Useful Concept for Understanding Journeys through Public Services? A Critical Interpretive Literature Synthesis. *Soc Policy Adm*. 2013;47(7):806-825. doi:10.1111/j.1467-9515.2012.00864.x | Social policy | public service |
| ^121^ Macklin J, Gullickson AM. What does it mean for an evaluation to be “valid”? A critical synthesis of evaluation literature. *Eval Program Plann*. 2022;91:102056. doi:10.1016/j.evalprogplan.2022.102056 | Research methodology | program evaluation |
| ^122^ MacMillan A, Gauthier P, Alberto L, Ives R, Williams C, Draper-Rodi DJ. The extent and quality of evidence for osteopathic education: A scoping review. *International Journal of Osteopathic Medicine*. March 2023:100663. doi:10.1016/j.ijosm.2023.100663 | Education | osteopathic training |
| ^123^ MacMillan A, Hohenschurz-Schmidt D, Migliarini DV, Draper-Rodi DJ. Discrimination, bullying or harassment in undergraduate education in the osteopathic, chiropractic and physiotherapy professions: A systematic review with critical interpretive synthesis. *International Journal of Educational Research Open*. 2022;3:100105. doi:10.1016/j.ijedro.2021.100105 | Higher education | discrimination, bullying or harassment in undergraduate education |
| ^124^ Malik S, Gunn S, Robertson N. The Impact Of Patient Suicide on Doctors and Nurses: A Critical Interpretive Meta-Synthesis. *Arch Suicide Res*. 2022;26(3):1266-1285. doi:10.1080/13811118.2021.1885533 | Health- Mental health | suicide |
| ^125^ Malla C, Aylward P, Ward P. Knowledge translation for public health in low- and middle- income countries: a critical interpretive synthesis. *Glob Health Res Policy*. 2018;3:29. doi:10.1186/s41256-018-0084-9 | Knowledge translation | knowledge translation for public health |
| ^126^ Mannell J, Lowe H, Brown L, et al. Risk factors for violence against women in high-prevalence settings: a mixed-methods systematic review and meta-synthesis. *BMJ Glob Health*. 2022;7(3). doi:10.1136/bmjgh-2021-007704 | Welfare | risk factors for violence against women in high-prevalence settings |
| ^127^ Markoulakis R, Kirsh B. Difficulties for University Students with Mental Health Problems: A Critical Interpretive Synthesis. *Rev High Ed*. 2013;37(1):77-100. doi:10.1353/rhe.2013.0073 | Health- Mental health | mental health of university students |
| ^128^ Martinus Hauge A, Otto EI, Wadmann S. The sociology of rationing: Towards increased interdisciplinary dialogue - A critical interpretive literature review. *Sociol Health Illn*. 2022;44(8):1287-1304. doi:10.1111/1467-9566.13507 | Health- Health equity and public health | healthcare rationing |
| ^129^ Masson M, Knouzi I, Arnott S, Lapkin S. A Critical Interpretive Synthesis of Post-Millennial Canadian French as a Second Language Research across Stakeholders and Programs. *The Canadian Modern Language Review*. 2021;77(2):154-188. doi:10.3138/cmlr-2020-0025 | Education | Canadian French as a Second Language (FSL) |
| ^130^ Mathieson A, Grande G, Luker K. Strategies, facilitators and barriers to implementation of evidence-based practice in community nursing: a systematic mixed-studies review and qualitative synthesis. *Prim Health Care Res Dev*. 2019;20:e6. doi:10.1017/S1463423618000488 | Health- Nursing and midwifery | implementation of evidence-based practice |
| ^131^ Mattison CA, Lavis JN, Wilson MG, Hutton EK, Dion ML. A critical interpretive synthesis of the roles of midwives in health systems. *Health Res Policy Syst*. 2020;18(1):77. doi:10.1186/s12961-020-00590-0 | Health- Nursing and midwifery | roles of midwives in health systems |
| ^132^ Mattison C, Bourret K, Hebert E, et al. Health systems factors impacting the integration of midwifery: an evidence-informed framework on strengthening midwifery associations. *BMJ Glob Health*. 2021;6(6). doi:10.1136/bmjgh-2020-004850 | Health- Nursing and midwifery | strengthening midwifery associations |
| ^133^ McDonough BA. Critical Information Literacy in Practice: An Interpretive Synthesis [Doctoral dissertation]. 2014. | Literacy | information literacy |
| ^134^ McFerran KS, Garrido S, Saarikallio S. A critical interpretive synthesis of the literature linking music and adolescent mental health. *Youth Soc*. 2016;48(4):521-538. doi:10.1177/0044118X13501343 | Health- Mental health | linking music and mental health |
| ^135^ McFerran KS, Lai HIC, Chang W-H, et al. Music, rhythm and trauma: A critical interpretive synthesis of research literature. *Front Psychol*. 2020;11:324. doi:10.3389/fpsyg.2020.00324 | Music therapy | rhythm and trauma |
| ^136^ McIntyre L, Tougas D, Rondeau K, Mah CL. “In”-sights about food banks from a critical interpretive synthesis of the academic literature. *Agric Human Values*. 2016;33(4):843-859. doi:10.1007/s10460-015-9674-z | Social policy | food banks |
| ^137^ McIsaac J-L, Warner G, Lawrence L, et al. The application of implementation science theories for population health: A critical interpretive synthesis. *AMS Public Health*. 2018;5(1):13-30. doi:10.3934/publichealth.2018.1.13 | implementation science | population health |
| ^138^ McKevitt S, White M, Petticrew M, et al. Typology of how “harmful commodity industries” interact with local governments in England: a critical interpretive synthesis. *BMJ Glob Health*. 2023;8(1). doi:10.1136/bmjgh-2022-010216 | Health- Healthcare delivery, policy and research | harmful commodity industries and non-communicable diseases risk |
| ^139^ McKibbin G, Humphreys C, Hamilton B. Prevention-enhancing interactions: a Critical Interpretive Synthesis of the evidence about children who sexually abuse other children. *Health Soc Care Community*. 2016;24(6):657-671. doi:10.1111/hsc.12260 | Psychology | prevention of sexual abuse by children against other children |
| ^140^ Meredith C, McKerchar C, Lacey C. Indigenous approaches to perinatal mental health: a systematic review with critical interpretive synthesis. *Arch Womens Ment Health*. 2023;26(3):275-293. doi:10.1007/s00737-023-01310-7 | Health- Mental health | perinatal mental health |
| ^141^ Moat KA, Lavis JN, Abelson J. How contexts and issues influence the use of policy-relevant research syntheses: a critical interpretive synthesis. *Milbank Q*. 2013;91(3):604-648. doi:10.1111/1468-0009.12026 | Health- Healthcare delivery, policy and research | influence of context- and issue-related factors |
| ^142^ Montesanti RS. The Participation of Marginalized Populations in Health Services Planning and Decision Making [Doctoral dissertation]. 2014. | Health- Healthcare delivery, policy and research | community participation |
| ^143^ Morgan H. ‘Pushed’ self-tracking using digital technologies for chronic health condition management: a critical interpretive synthesis. *Digit Health*. 2016;2:205520761667849. doi:10.1177/2055207616678498 | Health- Healthcare delivery, policy and research | digital technologies for chronic health |
| ^144^ Morgan HM, Entwistle VA, Cribb A, et al. We need to talk about purpose: a critical interpretive synthesis of health and social care professionals’ approaches to self-management support for people with long-term conditions. *Health Expect*. 2017;20(2):243-259. doi:10.1111/hex.12453 | Health- Clinical health services | self-management of chronic conditions |
| ^145^ Morgan RL, Kelley L, Guyatt GH, Johnson A, Lavis JN. Decision-making frameworks and considerations for informing coverage decisions for healthcare interventions: a critical interpretive synthesis. *J Clin Epidemiol*. 2018;94:143-150. doi:10.1016/j.jclinepi.2017.09.023 | Health- Healthcare delivery, policy and research | coverage decisions for healthcare interventions |
| ^146^ Morrison LG, Yardley L, Powell J, Michie S. What design features are used in effective e-health interventions? A review using techniques from Critical Interpretive Synthesis. *Telemed J E Health*. 2012;18(2):137-144. doi:10.1089/tmj.2011.0062 | Health- Healthcare delivery, policy and research | design features of effective e-Health interventions |
| ^147^ Mothupi MC, Knight L, Tabana H. Measurement approaches in continuum of care for maternal health: a critical interpretive synthesis of evidence from LMICs and its implications for the South African context. *BMC Health Serv Res*. 2018;18(1):539. doi:10.1186/s12913-018-3278-4 | Health- Health equity and public health | continuum of care for maternal health in low-middle income countries |
| ^148^ Moyle W, Murfield J, Lion K. Therapeutic use of the humanoid robot, Telenoid, with older adults: A critical interpretive synthesis review. *Assist Technol*. April 2022:1-8. doi:10.1080/10400435.2022.2060375 | Health- Clinical health services | assistive technology older age |
| ^149^ Nguyen DTK, McLaren L, Oelke ND, McIntyre L. Developing a framework to inform scale-up success for population health interventions: a critical interpretive synthesis of the literature. *Glob Health Res Policy*. 2020;5:18. doi:10.1186/s41256-020-00141-8 | Health- Health equity and public health | scaling up population health interventions |
| ^150^ Nicholas DB, Attridge M, Zwaigenbaum L, Clarke M. Vocational support approaches in autism spectrum disorder: a synthesis review of the literature. *Autism*. 2015;19(2):235-245. doi:10.1177/1362361313516548 | Health- Health equity and public health | vocational support |
| ^151^ Nobels A, Vandeviver C, Beaulieu M, et al. “Too grey to be true?” sexual violence in older adults: A critical interpretive synthesis of evidence. *Int J Environ Res Public Health*. 2020;17(11). doi:10.3390/ijerph17114117 | Health- Health equity and public health | sexual violence in older adults |
| ^152^ Nunez J. An application of learning theories for STEM degree seeking students in community colleges [Doctoral dissertation]. 2018. | Education | learning theories for STEM students |
| ^153^ Pack R, Hilton G, Garcia-Bournissen F, Taylor T. Transforming possible risk into certain harm: A critical interpretive synthesis of the literature on perinatal cannabis use. *Contemp Drug Probl*. 2022;49(4):505-521. doi:10.1177/00914509221126549 | Health- Women's and maternal health | perinatal cannabis use |
| ^154^ Pahlman K, Fehross A, Fox GJ, Silva DS. Ethical health security in the age of antimicrobial resistance. *BMJ Glob Health*. 2022;7(1). doi:10.1136/bmjgh-2021-007407 | Health- Health equity and public health | health security antimicrobial resistance bioethics |
| ^155^ Patey AM, Hurt CS, Grimshaw JM, Francis JJ. Changing behaviour ’more or less’-do theories of behaviour inform strategies for implementation and de-implementation? A critical interpretive synthesis. *Implement Sci*. 2018;13(1):134. doi:10.1186/s13012-018-0826-6 | Implementation science | behaviour change |
| ^156^ Payne L. Continuity of care and its effect on patients’ motivation to initiate and maintain cardiac rehabilitation [Doctoral dissertation]. 2015. | Health- Clinical health services | continuity of care and patient motivation |
| ^157^ Perski O, Blandford A, West R, Michie S. Conceptualising engagement with digital behaviour change interventions: a systematic review using principles from critical interpretive synthesis. *Transl Behav Med*. 2017;7(2):254-267. doi:10.1007/s13142-016-0453-1 | Health- Healthcare delivery, policy and research | digital behaviour change interventions |
| ^158^ Pfeffer D, Wigginton B, Gartner C, Morphett K. Smokers’ understandings of addiction to nicotine and tobacco: A systematic review and interpretive synthesis of quantitative and qualitative research. *Nicotine Tob Res*. 2018;20(9):1038-1046. doi:10.1093/ntr/ntx186 | Health- Mental health | nicotine and tobacco |
| ^159^ Pinto JW, Bradbury K, Newell D, Bishop FL. Lifestyle and health behavior change in traditional acupuncture practice: A systematic critical interpretive synthesis. *J Altern Complement Med*. 2021;27(3):238-254. doi:10.1089/acm.2020.0365 | Health- Clinical health services | behaviour change within traditional acupuncture |
| ^160^ Plamondon KM, Caxaj CS, Graham ID, Bottorff JL. Connecting knowledge with action for health equity: a critical interpretive synthesis of promising practices. *Int J Equity Health*. 2019;18(1):202. doi:10.1186/s12939-019-1108-x | Health- Health equity and public health | knowledge with action for health equity |
| ^161^ Poirier MJP, Grépin KA, Grignon M. Approaches and alternatives to the wealth index to measure socioeconomic status using survey data: A critical interpretive synthesis. *Soc Indic Res*. 2020;148(1):1-46. doi:10.1007/s11205-019-02187-9 | Demographics | wealth index to measure socioeconomic status |
| ^162^ Pokharel B, Yelland J, Hooker L, Taft A. A systematic review of culturally competent family violence responses to women in primary care. *Trauma Violence Abuse*. 2023;24(2):928-945. doi:10.1177/15248380211046968 | Family violence | culturally competent models of care in the context of family violence |
| ^163^ Pooley EA, Beagan BL. The concept of oppression and occupational therapy: A critical interpretive synthesis. *Can J Occup Ther*. 2021;88(4):407-417. doi:10.1177/00084174211051168 | Occupational therapy | oppression and occupational therapy |
| ^164^ Prowse P-T, Nagel T. A Meta-Evaluation: The Role of Treatment Fidelity within Psychosocial Interventions during the Last Decade. *J Psychiatry*. 2015;18(2). doi:10.4172/Psychiatry.1000251 | Psychology | treatment fidelity |
| ^165^ Puurveen G, Baumbusch J, Gandhi P. From family involvement to family inclusion in nursing home settings: A critical interpretive synthesis. *J Fam Nurs*. 2018;24(1):60-85. doi:10.1177/1074840718754314 | Health- Nursing and midwifery | family involvement in nursing home settings |
| ^166^ Rakower J, Hallyburton A. Disease information through comics: A graphic option for health education. *J Med Humanit*. 2022;43(3):475-492. doi:10.1007/s10912-022-09730-9 | Health- Healthcare delivery, policy and research | health education using comics |
| ^167^ Ray H, Sobiech KL, Alexandrova M, Songok JJ, Rukunga J, Bucher S. Critical Interpretive Synthesis of Qualitative Data on the Health Care Ecosystem for Vulnerable Newborns in Low- to Middle-Income Countries. *J Obstet Gynecol Neonatal Nurs*. 2021;50(5):549-560. doi:10.1016/j.jogn.2021.05.001 | Health- Health equity and public health | ecosystem for vulnerable newborns in low-middle income countries |
| ^168^ Rega ML, Telaretti F, Alvaro R, Kangasniemi M. Philosophical and theoretical content of the nursing discipline in academic education: A critical interpretive synthesis. *Nurse Educ Today*. 2017;57:74-81. doi:10.1016/j.nedt.2017.07.001 | Health- Nursing and midwifery | nursing academic education |
| ^169^ Reid HA, Holmes JD, Laliberte Rudman D, Johnson AM. Representing informal caregivers of older adults in occupation-focused research: A critical interpretive synthesis. *Journal of Occupational Science*. 2023;30(3):472-486. doi:10.1080/14427591.2022.2122542 | Occupational therapy | occupational therapy informal caregiver |
| ^170^ Reidy C, Bracher M, Foster C, Vassilev I, Rogers A. The process of incorporating insulin pumps into the everyday lives of people with Type 1 diabetes: A critical interpretive synthesis. *Health Expect*. 2018;21(4):714-729. doi:10.1111/hex.12666. | Health- Clinical health services | incorporation of insulin pumps for people with Type 1 diabetes |
| ^171^ Reparon R, Block P, Fudge Schormans A, Laliberte Rudman D, Teachman G. Critiquing representations of intellectual disability in occupation-based literature. *Scand J Occup Ther*. 2024;31(1):2289897. doi:10.1080/11038128.2023.2289897 | Occupational therapy | occupational therapy representations of intellectual disability |
| ^172^ Risk J, Mohammadi L, Rhee J, Walters L, Ward PR. Barriers, enablers and initiatives for uptake of advance care planning in general practice: a systematic review and critical interpretive synthesis. *BMJ Open*. 2019;9(9):e030275. doi:10.1136/bmjopen-2019-030275 | Health- Clinical health services | advance care planning |
| ^173^ Rosic T, Lovell E, MacMillan H, Samaan Z, Morgan RL. Components of Outpatient Child and Youth Concurrent Disorders Programs: A Critical Interpretive Synthesis: Composantes des programmes de troubles concomitants des enfants et des jeunes ambulatoires : une synthèse interprétative critique. *Can J Psychiatry*. 2024;69(6):381-394. doi:10.1177/07067437231212037 | Health- Mental health | psychiatry outpatient child and youth concurrent disorders programs |
| ^174^ Salmon VE, Hay-Smith EJC, Jarvie R, et al. Implementing pelvic floor muscle training in women’s childbearing years: A critical interpretive synthesis of individual, professional, and service issues. *Neurourol Urodyn*. 2020;39(2):863-870. doi:10.1002/nau.24256 | Health- Women's and maternal health | maternal pelvic floor muscle exercising |
| ^175^ Sangrar R, Docherty-Skippen SM, Beattie K. Blended face-to-face and online/computer-based education approaches in chronic disease self-management: A critical interpretive synthesis. *Patient Educ Couns*. 2019;102(10):1822-1832. doi:10.1016/j.pec.2019.05.009 | Health- Healthcare delivery, policy and research | face-to-face and online/computer-based education for patients with chronic disease |
| ^176^ Schaaf M, Jaffe M, Tunçalp Ö, Freedman L. A critical interpretive synthesis of power and mistreatment of women in maternity care. *PLOS Glob Public Health*. 2023;3(1):e0000616. doi:10.1371/journal.pgph.0000616 | Health- Women's and maternal health | physical and verbal abuse and mistreatment of labouring women |
| ^177^ Schaaf M, Topp SM. A critical interpretive synthesis of informal payments in maternal health care. *Health Policy Plan*. 2019;34(3):216-229. doi:10.1093/heapol/czz003 | Health- Women's and maternal health | informal payments in maternal health care |
| ^178^ Schroerlucke D. Choke artists and clutch performers : a Critical Interpretive Synthesis [Doctoral dissertation]. 2014. | Psychology | performing under pressure conditions |
| ^179^ Schuster-Wallace CJ, Nouvet E, Rigby I, et al. Culturally sensitive palliative care in humanitarian action: Lessons from a critical interpretive synthesis of culture in palliative care literature. *Palliat Support Care*. 2022;20(4):582-592. doi:10.1017/S1478951521000894 | Health- Clinical health services | culturally sensitive palliative care in humanitarian action |
| ^180^ Schwartz C, Tooley L, Knight R, Steinberg M. Queering poppers literature: A critical interpretive synthesis of health sciences research on alkyl nitrite use and Canadian policy. *Int J Drug Policy*. 2022;101:103546. doi:10.1016/j.drugpo.2021.103546 | Health- Health equity and public health | gay men's health pharmaceutical use |
| ^181^ Shahram SZ, Smith ML, Ben-David S, Feddersen M, Kemp TE, Plamondon K. Promoting “zest for life”: A systematic literature review of resiliency factors to prevent youth suicide. *J Res Adolesc*. 2021;31(1):4-24. doi:10.1111/jora.12588 | Suicide | resiliency factors to prevent youth suicide |
| ^182^ Shore CB, Maben J, Mold F, Winkley K, Cook A, Stenner K. Delegation of medication administration from registered nurses to non-registered support workers in community care settings: A systematic review with critical interpretive synthesis. *Int J Nurs Stud*. 2022;126:104121. doi:10.1016/j.ijnurstu.2021.104121 | Health- Nursing and midwifery | delegation of medication administration |
| ^183^ Sinnott C, Georgiadis A, Park J, Dixon-Woods M. Impacts of operational failures on primary care physicians’ work: A critical interpretive synthesis of the literature. *Ann Fam Med*. 2020;18(2):159-168. doi:10.1370/afm.2485 | Health- Healthcare delivery, policy and research | operational failures on primary care physicians' work |
| ^184^ Steege R, Taegtmeyer M, McCollum R, et al. How do gender relations affect the working lives of close to community health service providers? Empirical research, a review and conceptual framework. *Soc Sci Med*. 2018;209:1-13. doi:10.1016/j.socscimed.2018.05.002 | Health- Healthcare delivery, policy and research | effect of gender relations of community health service providers |
| ^185^ Steele ME, Crooke AHD, McFerran KS. What about the teacher? A critical interpretive synthesis on literature describing music therapist/teacher consultation in schools. *Voices*. 2020;20(1). doi:10.15845/voices.v20i1.2839 | Music therapy | music therapist teacher support |
| ^186^ Stoyanov S, Kirschner PA. Text analytics for uncovering untapped ideas at the intersection of learning design and learning analytics: Critical interpretative synthesis. *J Comput Assist Learn*. 2023;39(3):899-920. doi:10.1111/jcal.12775 | Computer assisted learning | learning design and learning analytics intersection and synergy |
| ^187^ Strøm A, Slettebø T. Factors affecting user participation for nursing home residents with dementia: a critical interpretive synthesis. *European Journal of Social Work*. 2021;24(5):828-851. doi:10.1080/13691457.2021.1964442 | Social work | user participation nursing home residents with dementia |
| ^188^ Swain R, Forsyth F, Bowers B, et al. A critical interpretive synthesis of the lived experiences and health and patient-reported outcomes of people living with COPD who isolated during the COVID-19 pandemic. *Eur Respir Rev*. 2023;32(169). doi:10.1183/16000617.0031-2023 | Health- Clinical health services | lived experiences of people with COPD isolated at home during the COVID pandemic |
| ^189^ Swift E, O’Brien MR, Peters S, Kelly C. Healthcare professionals’ perceptions of pulmonary rehabilitation as a management strategy for patients with chronic obstructive pulmonary disease: a critical interpretive synthesis. *Disabil Rehabil*. 2022;44(4):520-535. doi:10.1080/09638288.2020.1769745 | Health- Clinical health services | pulmonary rehabilitation |
| ^190^ Sy MP, Carrasco R, Peralta-Catipon T, Yao DP, Dee V, Ching PE. Shedding light on hidden Filipino occupations as portrayed by mass media and scholarly resources: A critical interpretive synthesis. *Journal of Occupational Science*. March 2023:1-17. doi:10.1080/14427591.2023.2182348 | Occupational science | occupations as portrayed in the media |
| ^191^ Talseth A-G, Gilje FL. Nurses’ responses to suicide and suicidal patients: a critical interpretive synthesis. *J Clin Nurs*. 2011;20(11-12):1651-1667. doi:10.1111/j.1365-2702.2010.03490.x | Suicide | nurses responses to suicide |
| ^192^ Talseth A-G, Gilje FL. Liberating burdensomeness of suicide survivorship loss: A Critical Interpretive Synthesis. *J Clin Nurs*. 2017;26(23-24):3843-3858. doi:10.1111/jocn.13797 | Suicide | response to suicide by the people who are survived by them |
| ^193^ Talseth A-G, Gilje FL. Responses of persons at risk of suicide: A critical interpretive synthesis. *Nurs Open*. 2018;5(4):469-483. doi:10.1002/nop2.169 | Suicide | nursing understanding of patients at risk for suicide |
| ^194^ Tan SE, Kuschminder K. Migrant experiences of sexual and gender based violence: a critical interpretative synthesis. *Global Health*. 2022;18(1):68. doi:10.1186/s12992-022-00860-2 | Health- Health equity and public health | migrant experiences of sexual and gender based violence |
| ^195^ Tetui M, Zulu JM, Hurtig A-K, Ekirapa-Kiracho E, Kiwanuka SN, Coe A-B. Elements for harnessing participatory action research to strengthen health managers’ capacity: a critical interpretative synthesis. *Health Res Policy Syst*. 2018;16(1):33. doi:10.1186/s12961-018-0306-0 | Implementation science | participatory action research for health managers |
| ^196^ Thomas JD, Uwadiale AY, Watson NM. Towards equitable communication of kinesiology: A critical interpretive synthesis of readability research. *Quest*. 2021;73(2):151-169. doi:10.1080/00336297.2021.1897861 | Health- Healthcare delivery, policy and research | kinesiology plain language communication |
| ^197^ Tierney E, McEvoy R, O’Reilly-de Brún M, et al. A critical analysis of the implementation of service user involvement in primary care research and health service development using normalization process theory. *Health Expect*. 2016;19(3):501-515. doi:10.1111/hex.12237 | Health- Healthcare delivery, policy and research | service user involvement in research and development |
| ^198^ Tsimpida D, Kontopantelis E, Ashcroft DM, Panagioti M. Conceptual model of hearing health inequalities (HHI model): A critical interpretive synthesis. *Trends Hear*. 2021;25:23312165211002964. doi:10.1177/23312165211002963 | Health- Health equity and public health | hearing health inequalities |
| ^199^ Tuck CZ, Akparibo R, Gray LA, Aryeetey RNO, Cooper R. What influences cancer treatment service access in Ghana? A critical interpretive synthesis. *BMJ Open*. 2022;12(10):e065153. doi:10.1136/bmjopen-2022-065153 | Health- Clinical health services | access to and acceptance of cancer treatment |
| ^200^ Ulucanlar S, Lauber K, Fabbri A, et al. Corporate political activity: taxonomies and model of corporate influence on public policy. *Int J Health Policy Manag*. 2023;12:7292. doi:10.34172/ijhpm.2023.7292 | Public policy | unhealthy commodity industries corporate political activity |
| ^201^ Vélez CM, Wilson MG, Lavis JN, Abelson J, Florez ID. A framework for explaining the role of values in health policy decision-making in Latin America: a critical interpretive synthesis. *Health Res Policy Syst*. 2020;18(1):100. doi:10.1186/s12961-020-00584-y | Health- Healthcare delivery, policy and research | role of values in decision-making |
| ^202^ van Dongen SI, de Nooijer K, Cramm JM, et al. Self-management of patients with advanced cancer: A systematic review of experiences and attitudes. *Palliat Med*. 2020;34(2):160-178. doi:10.1177/0269216319883976 | Health- Clinical health services | self-management of cancer |
| ^203^ Waldboth V, Patch C, Mahrer-Imhof R, Metcalfe A. Living a normal life in an extraordinary way: A systematic review investigating experiences of families of young people’s transition into adulthood when affected by a genetic and chronic childhood condition. *Int J Nurs Stud*. 2016;62:44-59. doi:10.1016/j.ijnurstu.2016.07.007 | Health- Clinical health services | transition of children with chornic disease to adulthood |
| ^204^ Washa T. Mapping the life course trajectories of early life adversity: Toward a holistic model of developmental traumatology [Doctoral dissertation]. 2019. | Health- Mental health | trauma and early life adversity |
| ^205^ West R, Silverman MJ. Social Skills Instruments for Children with Autism Spectrum Disorder: A Critical Interpretive Synthesis. *J Music Ther*. 2021;58(2):121-154. doi:10.1093/jmt/thaa017 | Music therapy | social skills for children with Autism Spectrum Disorder |
| ^206^ White J, Bond C. The role that schools hold in supporting young people with selective mutism: a systematic literature review. *J Research in Spec Educ Needs*. 2022;22(3):232-242. doi:10.1111/1471-3802.12561 | Education | role of schools for supporting children with selective mutism |
| ^207^ Wilson E, Caswell G, Turner N, Pollock K. Managing medicines for patients dying at home: A review of family caregivers’ experiences. *J Pain Symptom Manage*. 2018;56(6):962-974. doi:10.1016/j.jpainsymman.2018.08.019 | Health- Clinical health services | informal caretakers at end-of-life |
| ^208^ Wilson AL, Jovanovic JM, Harman-Smith YE, Ward PR. A population health approach in education to support children’s early development: A Critical Interpretive Synthesis. *PLoS One*. 2019;14(6):e0218403. doi:10.1371/journal.pone.0218403 | Health- Health equity and public health | early childhood education planning |
| ^209^ Xu X, Xie J, Sun J, Cheng Y. Factors affecting authors’ manuscript submission behaviour: A systematic review. *Learn Pub*. 2023;36(2):285-298. doi:10.1002/leap.1521 | Academic publishing | selection of journal for academic publishing |
| ^210^ Yang Y, Boulton E, Todd C. Measurement of Adherence to mHealth Physical Activity Interventions and Exploration of the Factors That Affect the Adherence: Scoping Review and Proposed Framework. *J Med Internet Res*. 2022;24(6):e30817. doi:10.2196/30817 | Health- Healthcare delivery, policy and research | mHealth physical activity programs |
| ^211^ Yazdani S, Akbari Lake M, Ahmady S, Forootan A, Afshar L. Critical interpretive synthesis of the concept of value in medical education. *Res dev med edu*. 2015;4(1):31-34. doi:10.15171/rdme.2015.005 | Education | value in medical education |
| ^212^ Zhang L, Basham JD, Yang S. Understanding the implementation of personalized learning: A research synthesis. *Educational Research Review*. 2020;31:100339. doi:10.1016/j.edurev.2020.100339 | Education | implementation of personalized learning |
