# Supplementary material for: Making sense of conducting a critical interpretive synthesis: A scoping review
Source: Res Synth Methods. 2025 Oct 8;17(1):30–41. doi: 10.1017/rsm.2025.10041 (PMC12823206; doi:10.1017/rsm.2025.10041)
Supplement: Perlman et al. Supplementary Material 2 — Perlman et al. supplementary material [file S1759287925100410sup002.pdf]

Data Extraction Form (copied from Covidence)

MANUSCRIPT

- **Author(s)**
- **Manuscript title**

  Title of paper / abstract / report that data are extracted from
- **Year of publication**

  i.e., year of publication, journal, volume, issue, pages, language, status (reviewed, accepted, etc.)
- **Journal**
- **Volume: Issue**
- **Pages**

STUDY DESIGN

- **Country**
- Country in which the research took place
- **Research context**
- What is the area of research?

APPLICABILITY

- **Topic area**

  e.g., health policy, management

  e.g., chronic disease, nutrition

  BE SPECIFIC
- **CIS reasoning**

  Why was a CIS design chosen?
- **Articles in CIS**

  How many articles were included in the CIS?
- **Question or topic?**
- **How many questions asked?**
- **Dixon-Woods Phase 1: Review question**

  What is the nature of the review question? e.g., stagnant, evolving? broad? specific?
- **Dixon-Woods Phase 2: Literature Search**

  What search strategies were used?
- **How were articles chosen to be included?**
- **Dixon-Woods Phase 3: Sampling**

  Was sampling mentioned? What type of sampling used? How was the sampling informed?

- **Dixon-Woods Phase 4: Determination of Quality**

  Was there quality assessment? At what stage? Were low quality papers included?
- **Dixon-Woods Phase 5: Data Extraction**

  What method was used to extract data?
- **Were appraisal and extraction performed/reported together?**
- **Dixon-Woods Phase 6: Interpretive Synthesis**

  How is the synthesizing argument generated? Are synthetic constructs/third order constructs generated? Does the synthesizing argument include critique of existing evidence?
- **Were data extraction and synthesis performed/reported together?**

FACTORS RELATED TO QUALITY OF RESEARCH

- **Limitations**

  What are the limitations reported as related to utilization of the study design?
- **Strengths**

  What are the strengths reported as related to utilization of the study design?
- **Contextual considerations**

  What is the interaction between the study design subject of interest?

REPORTING DETAILS

Was a PRISMA used?

1. Yes
2. No

Was a protocol published?

1. Yes
2. No

NOTES?
